# Applications of In Vitro Tissue Culture Technologies in Breeding and Genetic Improvement of Wheat

**DOI:** 10.3390/plants11172273

**Published:** 2022-08-31

**Authors:** Akila Wijerathna-Yapa, Vinita Ramtekey, Buddhini Ranawaka, Bhoja Raj Basnet

**Affiliations:** 1 ARC Centre of Excellence for Plant Success in Nature and Agriculture, St Lucia, QLD 4072, Australia; 2 School of Biological Sciences, The University of Queensland, St Lucia, QLD 4072, Australia; 3 ICAR-Indian Institute of Seed Science, Kushmaur, Mau, Uttar Pradesh 275103, India; 4 Centre for Agriculture and the Bioeconomy, Institute for Future Environments, Queensland University of Technology (QUT), 2 George Street, Brisbane, QLD 4000, Australia; 5 Global Wheat Program, International Maize and Wheat Improvement Center (CIMMYT), El Batán 56237, Mexico

**Keywords:** wheat biotechnology, tissue culture, genome editing, CRISPR/Cas

## Abstract

Sources of new genetic variability have been limited to existing germplasm in the past. Wheat has been studied extensively for various agronomic traits located throughout the genome. The large size of the chromosomes and the ability of its polyploid genome to tolerate the addition or loss of chromosomes facilitated rapid progress in the early study of wheat genetics using cytogenetic techniques. At the same time, its large genome size has limited the progress in genetic characterization studies focused on diploid species, with a small genome and genetic engineering procedures already developed. Today, the genetic transformation and gene editing procedures offer attractive alternatives to conventional techniques for breeding wheat because they allow one or more of the genes to be introduced or altered into an elite cultivar without affecting its genetic background. Recently, significant advances have been made in regenerating various plant tissues, providing the essential basis for regenerating transgenic plants. In addition, *Agrobacterium*-mediated, biolistic, and *in planta* particle bombardment (iPB) gene delivery procedures have been developed for wheat transformation and advanced transgenic wheat development. As a result, several useful genes are now available that have been transferred or would be helpful to be transferred to wheat in addition to the current traditional effort to improve trait values, such as resistance to abiotic and biotic factors, grain quality, and plant architecture. Furthermore, the *in planta* genome editing method will significantly contribute to the social implementation of genome-edited crops to innovate the breeding pipeline and leverage unique climate adaptations.

## 1. Introduction

Food security is one of the most significant challenges facing the future of our planet. By 2050, the world will need nearly double the amount of food produced today to feed an expected nine billion people. To achieve this goal effectively, food production must be significantly increased sustainably on existing arable land while addressing the challenges posed by climate change. Crop breeding programs and improved management regimes have led to steady increases in crop yields over the past five decades. However, the rate of yield improvement has plateaued [1]. The relatively small annual incremental gains in yield (wheat yields increasing at 0.9% per year, non-compounding rates; at these rates, global production increases by ∼38%) are not sufficient to meet projected demands by 2050. Therefore, producing better, higher-yielding crops is the ideal approach to be explored immediately [2].

The genetic improvement of wheat has traditionally been achieved through sexual hybridization between related species, resulting in numerous cultivars with high yields and superior agronomic performance. Conventional plant breeding, sometimes combined with classical cytogenetic techniques, continues to be the primary method of cereal crop improvement [3]. Given the worldwide predominance of cereal grains in the human diet, cereal crops quickly emerged as prime targets for improvement by genetic transformation. Wheat genetic processing technology has progressed rapidly during the last decade. Initially, the genetic transformation of cereals was based on the introduction of DNA into protoplasts and subsequent callus production for the regeneration of fertile plants. The application and prospects of plant tissue culture and transformation technology in wheat for introducing resistance against fungal and viral diseases and abiotic constraints and improving nutritional quality are reviewed in this paper.

The maintenance of genetic diversity is essential in a breeding program to ensure sustainable production. Plant breeders have extensively leveraged genetic variation from different gene pools to improve genetic diversity. Hence there is a need to look for an alternative advanced and cost-effective strategy for genetic enrichment of the gene pool and allelic diversification to overcome the limitations of narrow and uniform genetic variations [4]. As a strategy, integrating tissue culture techniques with plant biotechnology and breeding programs offers significant potential for increasing crop genetic diversity. Many years ago, these strategies were exploited to manipulate genetic variability and create genetic diversity to enrich the available genetic pool and make it desirable for a plant breeder to use for crop improvement [5]. Plant tissue culture includes a culture of the cell protoplast, anther and microspore (immature pollen grain), ovary and ovules, and embryo, which features genetic and epigenetic variation in the breeding material. Such in vitro culture methods exploit all the available genetic variability and reduce the period of the breeding program to develop tolerant and resistant genotypes [6]. Primarily, plant tissue culture is used in vegetatively propagated crops and self-pollinated crops, especially with narrower genetic bases. As an example, being an autogamous crop, wheat potentially possesses a narrow genetic base as the chances of the natural generation of genetic variation are about 3–5% due to its rare outcrossing actions. Therefore, in vitro techniques can be a potential solution for manipulating the desired trait, enriching the genetic base, and recovering desirable variation [7].

Physical and chemical mutagens, epigenetic agents such as DNA demethylases, and histone deacetylase inhibitors, in combination with in vitro techniques, are most frequently used for genetic rearrangement and epigenetic reprogramming through the induction of mutations, DNA and histone methylation, and histone acetylation [8,9]. Likewise, the advanced genome editing approach, along with plant tissue culture and Agrobacterium transformation, has emerged as the most promising alternative for the genetic manipulation of traits of interest [8,10]. CRISPR/Cas9 nuclease-mediated genome editing can precisely edit genes or any part of the plant genome to improve critical agronomic traits. However, traditional wheat breeding can achieve the same goal, but it can take up to 7–10 years compared to seeing the benefits of CRISPR technology in considerably less time [11].

In this regard, breeding new wheat varieties to cope with biotic and abiotic stresses represents one of the breeders’ significant challenges. For decades, breeding strategies have included selection, hybridization, mutation induction using chemical and physical agents, and somaclonal variation (Table 1). More recently, genome editing technologies, the availability of whole genome sequences, efficient tissue culture, and transformation methodologies have, remarkably, been able to facilitate wheat breeding.

## 2. Intraspecific Hybridization

When the crosses between lines belong to groups with different subspecies or ecotypes from the same species, termed intraspecific hybridization, it increases the population’s genetic diversity [12]. Instantly, a low-cost and high-throughput approach such as in vitro techniques made it feasible to improve the genetic base without crossing, cost-effectively, with a need for less time and space. Somatic intraspecific hybridization, induced via protoplast fusion and subsequent polyploidization between *japonica* rice (*Oryza sativa* L. subsp *japonica*) and *indica* rice (*Oryza sativa* L. subsp *indica*), led to genetic and epigenetic changes at the early stage of hybrid formation and development due to genomic shock or genomic perturbation [13]. Another study showed intraspecific hybridization among the five wheat genotypes in all possible combinations to achieve the desired level of genetic variation [14]. Significant genetic variability was observed for morphological and physiological traits, such as plant height, spike length, spike density, grain weight/spike, and more, under field conditions.

Likewise, intraspecific hybridization enabled the study of the inheritance pattern and heterosis rate of different quantitative traits such as plant height, productive tiller capacity, and internode length in winter soft wheat [15]. However, significant genetic variation was exhibited regarding grain spike/plant, grain yield/plant, and seed index at a 0.01% significance level in intraspecific hybrids and their F2 generation of bread wheat [16]. In the case of winter rye, the intraspecific hybridization approach is used as a source of the creation of intrapopulation genetic variability. The intraspecific hybridization in the reciprocal and backcrossing method increases intrapopulation genetic variability for quantitative traits [17]. A little phenotypic variability was observed at a 5% significance level in the F1 hybrid related to yield regeneration and 1000 grain weight in winter rye. Even though intraspecific hybrids are closely related, parental genomes belonging to the same species exhibit a different pattern of heterosis due to the unique genetic interaction of alleles and epialleles [18]. Such a successful hybridization can enrich the genetic base by creating the genetic source material in the breeding program.

## 3. Interspecific and Intergeneric Hybridization

Interspecific hybridization occurs when crosses are made between different cultivated species belonging to the same genus. In contrast, the outcome of the combination of a distinct genus (cultivated species with their wild relatives) is known as intergeneric hybridization. These two approaches are the critical driving force in generating a different combination of hybrid lines, such as synthetic amphiploid lines, alloplasmic lines, and alien gene introgression lines, which act as a source of variation that leads to a broadening of the genetic variability and diversity of desired traits for crop improvement [19,20]. However, the success rate of interspecific and intergeneric hybridization is comparatively low compared to intraspecific hybridization due to cross-incompatibilities mainly related to pre- and post-fertilization barriers. To overcome these challenges, in vitro techniques utilizing somatic hybridization or embryo rescue came into the picture and have proven to be the best alternative. Several embryo rescue techniques such as embryo culture, ovary culture, ovule culture, anther culture, and protoplast culture protect embryos from successful hybridization and from premature abortion [13,21]. However, due to the genomic shock, this successful hybridization induces genetic and epigenetic modification at the early stages (zygote formation and development) of hybrids and successive generations [13]. Embryo rescue techniques such as immature embryo culture were used to develop an interspecific hybrid ACC between *B. napus* ‘Zhongshuang 9’ and *B. oleracea* ‘6m08’ [22].

The microspore-derived plants from developed hybrids exhibited higher genetic diversity in microspore-derived plants compared to both the parents by generating individuals with euploid, aneuploid, and unreduced gametes. Somatic hybridization can be induced via protoplast fusion following polyploidization between *japonica* rice and bread wheat (intergeneric hybridization) to analyze the genetic and epigenetic changes at the level of chromosomal elimination and the DNA sequence [13]. In both cases, the *japonica* rice protoplast was used as the recipient and suggested that these genomic changes in symmetric and asymmetric somatic hybrids resulted from the genomic shock induced at the early stage of the somatic hybrid. Intergeneric hybrids, developed by crossing hexaploid and tetraploid wheat with *Ae. Cylindrica*, followed by embryo rescue, improve the salt tolerance capacity in developed amphidiploid progenies [23]. Wide cross/intergeneric hybridization is made between rye and maize by following ovary culture to produce haploid embryos in the rye [24]. This study provides new possibilities for the introgression of genes through intergeneric hybridization in rye. However, a conventional embryo rescue method used to develop a haploid hybrid is problematic in auto-allogamous species.

A wheat–rice hybrid was created via an in vitro fertilization system, such as embryo culture, to overcome pre- and post-fertilization barriers in eliminating rice chromosomes at an early stage of zygote formation [25]. In this study, a different combination of alloplasmic zygote hybrids was observed. These wheat plants exhibited dwarf and infertile phenotypic behaviors. In the case of wheat, stripe and leaf rust is the most devastating disease to hamper its production. Wheat double haploid (DH) genetic stock was developed by the introgression of genes from *Imperata cylindrica* via embryo culture following colchicine treatment to produce a hybrid resistant to this fungal disease [26]. The developed DH lines exhibited better disease response and were more resistant against yellow and brown rust at seedling and adult plant stages than their parental lines. The identified resistant genes would be a valuable source of enrichment of the genetic base for resistance breeding and wheat improvement. Recently a suspension-derived protoplast fusion was performed to develop asymmetric somatic hybrids between bread wheat and *Agropyron elongatum* [27]. For plant regeneration in successive generations, ovary culture has been employed. The developed hybrids and progenies exhibited two different morphological characters: taller stems with large ears and grains.

In contrast, another type was short stems with strong tillering ability, and smaller ears and grains. Furthermore, GISH (genomic in situ hybridization) analysis showed the variation in somatic chromosome number ranges from 38 to 44, from which 70% of hybrid lines possess 2n = 42. This study suggested that asymmetric protoplast fusion could be a promising intergeneric hybridization tool. The chances of increasing genetic variation are much faster through interspecific and intergeneric hybridization than through intraspecific hybridization because the selected parental genotypes encounter diverse genetic backgrounds and regular interactions with enhanced sequence divergence [25].

### 3.1. Ph Locus

Another important discovery in wheat was the presence of the pairing homoeologous (Ph) locus, which enables the control of genetic recombination by suppressing meiotic pairing between wheat homeologs in interspecific and intergeneric hybridization [28]. Among all the identified Ph loci, Ph1 and Ph2 are the most important ones mapped on chromosome arm 5BL, 3DS, and 3AL, respectively, that effectively impose suppression of the homeolog pairing, which is not only restricted to wheat but its related species [29,30,31]. Looking into the importance of the Ph1 locus, a homoeologous pairing promoter gene, Hpp-5Mg (derived from *A. geniculata* with *T. aestivum*), promotes homoeologous recombination, and multiple crossovers between wheat and wild relative chromosomes led to an enrichment of the genetic base in pre-breeding materials [32]. However, suppression of the crossover in the hybrids between hexaploid wheat and wild relatives is possible when one of the parents carries a Ph1 deletion mutation. Furthermore, the screening of the Ph1 deletion mutant phenotype is cumbersome.

Further studies showed the *ZIP4 (TaZIP4-B2)* homologue in the Ph1 locus, exhibiting a high homoeologous chromosome level when crossed with wild relatives [33]. This suggested that the utilization of Tazip4-B2 mutants rather than complete Ph1 locus deletions would be much easier for alien gene introgression. The ph1b deletion line has been used to introgress powdery mildew resistance genes from *A. triuncialis* (5U) to bread wheat (5A) by inducing homoeologous meiotic pairing [34]. In addition to Ph1, a Ph2 locus encodes repair protein MSH7-3D, which plays a crucial role in the genomic and meiotic stabilization of allopolyploidy and paves the way for alien gene introgression from distantly related species of wheat to enrich the genetic diversity for substantial crop improvement [35]. This genetic control of meiotic homoeologous recombination has widely been used as a novel strategy in chromosome engineering technology to induce genetic variation and the introgression of desirable traits across wheat species [32]. 

### 3.2. Synthetic Wheat

At the dawn of the 20th century, to increase alien genetic variation, CIMMYT was emphasized on a wide crossing program by the utilization of synthetic hexaploid wheat as one of the parents in a distant hybridization or wide crossing program and with the utilization of translocation stock as well. The most significant breakthrough in wheat breeding came after developing the cultivar Veery ‘S’ from CIMMYT, which possesses the 1BL.1RS translocation from bread wheat and rye [36]. Later on, extensive efforts were made by CIMMYT to produce synthetic hexaploid wheat that combines the genomes of tetraploid and diploid distantly related wheat (*Ae. Tauschii*). A newly synthesized interspecific hybrid (*T. dicoccoides x Ae. Squarrosa* and their hybrid with *T. spelta and T. vulgure*) was developed through a union of unreduced gametes by artificial self-pollination and embryo culture followed by colchicine treatment for doubling the chromosome [37]. The developed synthetic hybrid was physiologically and phenotypically different from its parents and exhibited a wide range of essential and desirable variations in the segregating generation (F2, F3, and their offspring) in chromosome pairing and minor characteristic variation for agronomical traits, viz., plant height, ear length, awn, and many more. Similarly, crossing between elite *T. turgidum L*. s. lat. cultivars and *Ae. tauschii* accessions produced 1014 synthetic hexaploid combinations (2n = 6x = 42) through artificial hybridization, embryo rescue, and chromosome doubling of F1 hybrids [38]. This developed synthetic diversity by acting as a valuable source of beneficial alleles that are effectively being utilized in pre-breeding programs for alien gene transfer to broaden the genetic variability of the wheat gene pool at the DNA level through homology-directed introgression as well as the introduction of transcriptome shock that induces variation at RNA level [28,39]. Later on, the proportion of utilization of synthetic hexaploid wheat as a bridge species increased from 8–46% for the transfer of alien genes, and the addition and substitution of alien chromosomes linked to desirable traits from several wild relatives to bread wheat have enormously increased the genetic variability in the cultivated genotypes of wheat [40]. They have previously developed intergeneric hybrids among different alien genera of *Triticeae* (*Aegilops, Agropyron, Elymus, Haynaldia, Hordeum, Secale,* and *Triticum*) through controlled pollination, embryo rescue, embryo differentiation, and doubling of the chromosome [41]. The authors detected the involvement of 1B, 6B, and 5D satellited wheat chromosomes in the developed hybrids by somatic cytological investigation. Later, chromosome engineering transferred multiple alien segments into cultivated wheat that carry 7AL, 3BS, and 1AS from *Thinopyrum ponticum*, *Ae. longissimi* and *Triticum aestivum*, respectively [42]. The developed F1 hybrids and their translocated recombinant lines possess several valuable genes that enhance the genotypes’ genetic base and broaden genetic diversity. Similar results have observed the enhanced efficiency of alien gene transfer from *A. tauschii* segments to common wheat with the help of synthetic octoploid wheat by using the in vitro technique of embryo culture followed by chromosome doubling [43]. However, the wheat–rye 4R chromosome disomic addition line (WR35), through wide hybridization and embryo culture followed by chromosome doubling, exhibited resistance to multiple diseases [44]. The higher success rate of alien gene transfer followed by embryo culture or embryo rescue has overcome the limitation of reduced genetic variability [45]. Such synthetic interspecific and intergeneric hybridization paved the way for the introgression and incorporation of alien genes from distantly related genera.

## 4. In Vitro Culture in Doubled Haploid Production

“Haploid” refers to plants with gametic chromosomal numbers (n). A haploid is described as a doubled haploid (DH) when its number of chromosomes doubles [46]. For instance, DH wheat plants are developed when the gamete set of chromosomes (n) is doubled to preserve a balanced hexaploid genome. There are two main approaches to haploid induction: in vitro-based (IVB) and in vivo-based haploid induction. The IVB haploid induction relies on the development of immature male or female gametophytic cells, whereas in vivo haploid induction systems are based on inter/intraspecific hybridization and subsequent uniparental chromosome removal [9,47]. The most widely used IVB methods are in vitro-induced parthenogenesis (gynogenesis) and androgenesis (anther culture, isolated microspore culture, and shed microspore culture). Androgenesis has been identified as the most effective haploid induction method [48], and the fundamentals of various plant haploid induction techniques were discussed in some insightful reviews [5,9,47,49]. However, some essential crops such as tomato, pepper, and eggplant [50] and cereals such as wheat, rye, and barley [51] are recalcitrant to androgenesis. 

Once haploid plants are generated, their genomes must be doubled to establish a viable DH line. The homozygous nature of the DH plants’ genomes is crucial for accelerating the breeding process [52]. However, doubled haploids must be generated at a high frequency and in a reproducible manner to be effective in plant breeding programs. The efficacy and reproducibility of DH production are directly linked to the manipulation of critical elements involved in the process. For instance, optimizing culture media composition is crucial for cereal androgenesis because it determines the fate of the microspore, and it supplies nourishment throughout the induction of microspore embryogenesis and the regeneration phase. Diverse plant species and genotypes usually show different basal medium requirements to induce plant regeneration from microspores [53]. A few examples are the N6 medium for wheat, maize, and rice [54] and the W14 medium for oats [55].

## 5. Somaclonal Variation

The variation among the plants regenerated through any in vitro somatic cell or tissue culture is called somaclonal variation. It is an innovative and dynamic strategy for increasing genetic variety, broadening the genetic base, and, consequently, genetic enrichment to achieve significant and incremental crop improvement. [56,57]. It includes DNA-related genetic or epigenetic variations, which induce phenotypic changes distinguishable from the original parent. Primary causes include but are not limited to prolonged in vitro culturing, tissue culturing media composition, the presence of phytohormones, and certain other mechanical factors during culturing [58]. However, somaclonal variation has some complications related to in vitro cloning and the preservation of germplasm as the in vitro genetic alteration does not always give beneficial, positive, and stable effects.

**Figure 1:**
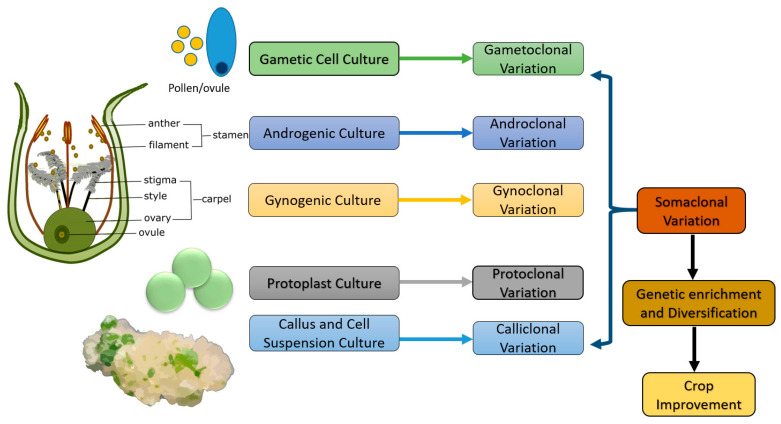
**Types of somaclonal variations.** Based on the tissue from which variation originates, somaclonal variation can be divided into the following types. **Gametoclonal variation:** variation observed among the plants regenerated from gametic cultures. **Androclonal variation**: observed among the plants regenerated from the anther (or) pollen culture. **Gynoclonal variation:** from ovule (or) ovary culture. **Protoclonal variation:** variation observed among the plants regenerated from protoplast cultures. **Calliclonal variation:** variation observed among the plants regenerated from callus cultures.

Furthermore, genotype dependency and genetic fidelity also reduce its applicability [8]. However, the plants regenerated from tissue culture are not true-to-type to the parent [59]. Phenotypic variation is most commonly observed in the crops under normal or stress conditions. Such unexpected and aberrant variations may be genetic or epigenetic, which arise due to gene mutation, chromosomal aberrations, genetic rearrangements, methylation, and transposable elements [60]. Genetic variations resulting from culture induction are heritable, whereas epigenetic variations are usually non-heritable, non-stable, and generally disappear when plants reproduce sexually. This genetic and epigenetic variation can be assessed by molecular tools such as molecular markers and reversed-phase HPLC (RP-HPLC). In addition, the methylation-sensitive Amplified Fragment Length Polymorphism (metAFLP) allowed simultaneous quantification of sequence modification and changes in DNA methylation patterns [58,61]. Different culture methods are also responsible for somaclonal variation in the regenerated plantlets. Based on the tissue from which the variation may arise, the somaclonal variation may be classified as gametoclonal, androclonal, gynoclonal, protoclonal, and calliclonal [62]. Below is a brief explanation of applying plant tissue culture or in vitro techniques to induce different types of variation to broaden and enrich the genetic base (Figure 1).

### 5.1. Gametoclonal Variation

The variation observed among the plants regenerated from in vitro culture of gametic cells is called gametoclonal variation. Such variations result from genetic recombination and are the core interest of plant breeders because of their simultaneous expression of dominant and recessive mutations [63]. Gametoclonal variation can be obtained through haploid production androgenic or gynogenic in vitro techniques. Haploids can be developed either through the male gametophyte (anther and microspore) or female gametophyte (ovules and ovary) via in vitro culture approaches known as androgenesis and gynogenesis, respectively [64]. However, these androgenic and gynogenic haploids are highly variable and susceptible to changes in ploidy level, gene shuffling, and mutation. Moreover, haploids are desirable and captivated by a plant breeder because of a single allelic gene present in a chromosome, which leads to the expression of traits governed by a recessive gene [63]. Therefore, genetic manipulation and manifestation become easy and are able to induce mutation and enhance the genetic background of crops.

Furthermore, the single recombination and the chromosomal instability to pair during meiosis make haploids a potential tool for a broad crossing program for introducing a novel gene of interest with reduced time cost-effectively. For example, a significant chromosomal variation in ring and rod bivalent formations was observed among the gametoclones generated from bread wheat cultivars, namely Sids 5 and Giza 163 [65]. Likewise, a significant genetic variation for different agronomical traits in R5 and R6 gametoclones of 15 genotypes of wheat under different sowing times was observed by performing field evaluations and inter-simple sequence repeat (ISSR) marker analysis [66]. In addition, a better heat stress tolerance capacity of gametoclones was observed. Similarly, analysis of the gametoclonal variability for vegetative, morphological, and biological traits exhibited a wide range of genetic variation in the doubled haploids (DH) androgenic rice lines against blast resistance [67]. These studies prove that gametoclonal variation could be used as a genetic resource in breeding programs for crop improvement and different biotic and abiotic stress tolerance.

### 5.2. Androclonal Variation

The in vitro culturing of anthers containing immature pollen grains or microspores on a nutrient medium is called anther and microspore culture. Such a type of culture technique results in androclonal variations. This is the most common, effective, and highly sensitive approach for haploid production [9]. Each plant produced from a single cell (microspore) is subjected to genetic shuffling and a chromosomal aberration in the anther culture. Consequently, this creates a diverse genotypic group that ultimately broadens the genetic diversity and variability in the population within a short period, increasing selection efficiency [68,69]. There are reports on the successful application of anther culture in developing haploid somatic cells in wheat and maize that can be utilized differently to create genetic variability [64]. Interspecific/intergeneric hybrids are a base for thousands of pollen grains with diverse genotypic groups and are more responsive to anther culture. This single genetic combination of haploids can be beneficial in selecting plants with desirable traits.

Furthermore, a significant genetic variation for all in vitro-studied traits among the genotypes and their hybrids was developed through anther culture in bread wheat under different drought stress treatments [70]. Moreover, this study also suggested that the androgenic plants developed from F_1_ hybrids are more responsive to anther culture than the plants regenerated from parents. Therefore, F_1_ hybrids can be the best option for haploid development to create variation and genetic enrichment via a positive heterotic effect. However, the genotype and species specificity and the formation of a higher frequency of albino plants limit the use of anther culture in plant breeding. Similar results were obtained in Asian cultivated rice and wheat [69,71]. Allele segregation with varying ploidy levels (from 2x to 6x) and somaclonal variation were observed in plants generated using anther cultures of hybrids in the Latvian wheat breeding program. Looking at the value of doubled haploid lines in accelerating a wheat breeding program, it is important to note that anther and microspore culture methods need to be optimized in the future for better results, i.e., genotype and species independent [69].

### 5.3. Gynoclonal Variation 

The in vitro culturing of an unpollinated female gametophyte (ovary, ovule, and flower buds) on a suitable solid or liquid nutrient medium is called ovary and ovule culture. The variations observed due to the tissue culture approach are called gynoclonal variations. In vitro gynogenesis is a second method of haploid production used in several agriculturally essential crops such as maize, barley, and wheat [72]. This method has proven significant in overcoming the limitation of androgenesis in producing a higher ratio of albino plants and male-sterile genotypes [6,73,74]. Furthermore, the in vitro gynogenesis method is used with salt stress (100 mM) during callus proliferation to create genetic variability among regenerated plantlets to permit the selection of salt-tolerant lines derived from durum wheat [75]. Further to this, they reported that among different haploid production methods, gynogenesis efficiency was at a par with interspecific crosses and isolated microspore culture [76]. They also observed that the plants regenerated from gynogenesis and crosses were green compared to isolated microspore culture, where most plants were albino. Therefore, despite it being a less-used method, gynogenesis has proved to be a successful green haploid plant production approach, which can be further used in the durum wheat breeding program. Likewise, testing the salt-stressed durum wheat genotypes under field conditions, they observed the genetic variation in a difference in ploidy level of plants regenerated through gynogenesis [76]. They also suggested that genotypes with a significant haploid induction rate do not necessarily have a good capacity for haploid plantlet production. Therefore, there is a need to optimize the gynogenesis protocol to fix different agronomic traits such as plant height, panicle length, spikelet number, seed size, and haploid production rates to overcome the genotype specificity. In addition to this, the callus formation using gynogenic culture provides the probability of gametoclonal variation in nature.

### 5.4. Protoclonal Variation

The variation that occurs through protoplast culture is known as protoclonal variation [77]. Furthermore, through induced variation and genetic engineering, protoplast culture has improved several agronomical and genetic traits such as disease resistance and biotic and abiotic stress tolerance [78]. Henceforth, it provides a novel and quick approach to creating genetic variation and the introgression of an alien gene into domesticated lines to enhance genetic gain.

A plant cell without a cell wall is a protoplast, which can dedifferentiate and follow the normal mitotic division to undergo chromosomal rearrangement by developing into a callus and different organs during tissue culture. Protoplast fusion is the physical process where two or more protoplasts come into contact with each other in the presence of some fusion-inducing agents such as polyethylene glycol [79]. Henceforth, a protoplast fusion can act as a practical plant breeding tool to transfer desirable and valuable traits such as disease resistance, and biotic and abiotic tolerance, thereby creating interspecific and intergeneric hybrids as well as cybrids. For example, a morphological variation was observed associated with genetic variability among the hybrids developed by the tri-parental protoplast fusion of *Brassica* sp. [80]. The developed hybrids were classified into 12 distinct groups based on their morphology variations and were confirmed by flow cytometry, genomic in situ hybridization, and molecular markers. This study suggested that high variability may help broaden Brassica’s existing gene pool, and the protoplast fusion technique can be further used to enrich the genetic base. Similarly, another study explains the importance of protoplast fusion and somatic hybridization to gene transfer and creating new genetic variations among sexually incompatible plant species to widen the genetic base and diversity [81]. Furthermore, the use of the transient transformation and genome editing of plant protoplasts may help integrate foreign DNA into the genome and thereby rearrange the genome to create desired variation and utilization of these variations for crop improvement [82].

### 5.5. Calliclonal Variation 

The plant variation observed due to callus and cell suspension culture is called calliclonal variation. Somatic embryogenesis by callus and cell suspension culture is an alternative approach to producing a calliclonal variation. Generally, the older callus and cell suspension cultures produce a higher number of calliclonal variants than fresh tissues because of the augmented mutation rate [8]. The plants regenerated from callus/cell suspension cultures are transferred to field/glasshouse conditions and can be directly screened for desirable traits/variation, whereas in vitro selection at a cellular level can be carried out by their ability to grow in the presence of an inhibitor for some specific traits such as antibiotic resistance or biotic/abiotic tolerance [56,59]. However, phenotypic variation in selfed progenies of a tissue culture originated a stable line of rice named TC-reg-2008 compared to its wild type under several abiotic stresses [59]. This phenotypic variation was associated with cryptic heritable genomic changes, distributed across the 12 chromosomes, and affected many functional genes.

These heritable genomic modifications can generate genetic diversity and enrich the genetic base due to the induction of single nucleotide polymorphisms (SNPs), base substitution, transposable elements, and its methylation under tissue culture practices. A cell suspension culture can be used to produce plantlets in sugarcane and observe the phenotypic variation concerning proliferation, and the shoot and root regeneration response [83]. Random amplified polymorphic DNA (RAPD) and ISSR confirmed this phenotypic variation, which detected the presence of genetic variation and diversity among the plantlets. In the same way, several researchers have observed morphological and phenotypical variations in plants regenerated through somatic embryogenesis in different agriculturally essential crops such as wheat, barley, and *Cymbopogon winterianus*. The embryogenic callus of maize from immature embryos was used to study the whole genome profiling of DNA methylation [84,85,86,87]. Interestingly, they recognized that DNA methylation losses are more common than gains of DNA methylation in the callus, primary regenerated plants, and their selfed progenies. These losses are responsible for producing an epigenetic footprint, i.e., somatically heritable variation arises during the regeneration process of selfed progenies compared to primary plantlets.

The mature embryo is rapidly recognized as a valuable alternative to the immature embryo for plant regeneration. Given this, mature embryo cultures of all agronomically valuable wheat genotypes for molecular assessment of the morphogenic pathway and study showed the genotype-dependent differences at different developmental stages throughout plant regeneration [88]. Likewise, genetic and epigenetic variations were assessed with the help of metAFLP [89]. They reported the sequence and DNA methylation changes in triticale plants regenerated through embryo culture.

## 6. Mutation Breeding

The premise of mutation breeding is to use exogenous agents to cause heritable changes to DNA. Exposure to physical or chemical factors causes alterations in plant cells. A sudden heritable alteration in the genetic material of an organism either spontaneously or artificially refers to mutation. The chances of desirable spontaneous mutation are scarce, i.e., 10^−6^. Therefore in vitro-induced mutagenesis with the help of physical (e.g., X-rays, gamma rays, cosmic rays) and chemical mutagens (e.g., ethyl methyl sulphonate (EMS), ethyl-ethyl sulphonate (EES), ethyleneimine (EI)) are the best alternatives to change the genomic background to increase the frequency of desirable genes or alleles [90]. However, in vivo and in vitro random physical and chemical mutagenesis could generate specific mutation types, artificially increasing the genetic diversity of agronomically important traits in plant breeding programs at a higher frequency than spontaneous mutation [91].

Any plant tissue can be mutagenized, and DNA alterations can be induced by exposing it to specific conditions. However, gametes (anther and ovary) are the most preferred as they are easy to target and generate heritable gametoclonal variation. Three types of mutagens, namely gamma radiation, laser rays, and EMS at various doses, treatments, and concentrations, play a significant role in inducing functional genetic variability in bread wheat to improve yield-contributing traits compared to their parent cultivars [92]. Later, combined mutation with salinity screening was performed to assess in vitro rice plantlets (White ponni and BPT-5204) generated from seed cultures to produce somaclonal variation in and selection of salt-tolerant callus lines [93]. To investigate the effect of in vitro chemical mutagenesis, different concentrations of EMS were used on the embryonic calli of the Kavuni cultivar of black rice for characteristics associated with semi-dwarfism, and this suggested that a 0.05% concentration is optimum for the creation of novel somaclonal variation related to morphological and biochemical traits [94]. For instance, a gamma-ray-induced in vitro mutagenesis in embryonic sugarcane callus culture [95]. They observed that this method paves the way for the generation of superior mutants that enhance genetic variation. Furthermore, chemical mutagens such as 2,4-D and EMS on in vitro plantlets of sugarcane cultivar (Co86032) were generated from young leaf rolls culture to produce a somaclonal variation that is to be utilized in a breeding program [96].

Recently, TILLING (Targeting Induced Local Lesions IN Genomes), a well-known reverse genetic approach in combination with a chemical mutagen, has been providing a rapid discovery and high frequency of random point mutations in the plant genome, which has led to the production of a range of phenotypic and genotypic variants [97]. By looking into the importance of TILLING, a study combined EMS mutagenesis with high-throughput genome-wide screening to detect the point mutation in a mutagenized population developed from the mature seed-derived *calli* of rice [98]. This study observed the different phenotypic and genotypic variants in heterozygous and homozygous mutant lines. Similarly, site-directed mutagenesis has been facilitating the new plant breeding program with the help of genome editing tools such as clustered regularly interspaced short palindromic repeats (CRISPR), transcription activator-like effector nucleases (TALEN), zinc-finger nucleases (ZFN), and oligonucleotide-directed mutagenesis (ODM), inducing genetic variability and diversity in the original genomic background [99]. Therefore mutation breeding can be the most promising alternative to conventional breeding for genetic enrichment and diversification.

**Table 1:** Methods used for in vitro techniques for crop improvement.

Crop	In Vitro Techniques	Function/Traits	References
**Interspecific hybridization**			
Pea (*P. sativum* × *P. fulvum*)	Immature embryo culture	Interspecific hybrid	[100]
Brassica (*B. napus* × *B. rapa*)	Embryo rescue via ovule culture	Interspecific hybrid	[101]
Rice (*Oryza sativa* × *Oryza australiensis*)	Young embryo rescue	Interspecific hybrid	[102]
Lentil (*Lens culinaris* × *Lens tomentosus*)	Ovule rescue technique	Interspecific hybrid between	[103]
Barley (diploid and tetraploid domestic barley × tetraploid wild barley)	Immature embryo culture	Induces genetic variation	[104]
Wheat (*T. aestivum* × *Dasypyrum villosum*)	Embryo rescue	Induces powdery mildew resistance	[105]
Wheat (*T. aestivum* × *Dasypyrum villosum*)	Embryo rescue	Induces stem rust resistance gene UG99	[106]
*Aegilops tauschii* × *Triticum aestivum* *Aegilops ovata* × *T. aestivum* *Aegilops cylindrica* × *T. aestivum* *Aegilops speltoides* × *T. aestivum*	Embryo culture	Alien gene introgression	[107]
*T. dicoccum* × *T. timopheevii*	Immature embryo culture	Creation of genetic variation	[108]
**Intergeneric hybridization**			
Rice (*Oryza sativa* × *Leersia perrieri*)	Embryo rescue	Intergeneric hybrid	[109]
Wheat × Barley (*Triticum aestivum* L. × *Hordeum vulgare* L.)	Embryo rescue	Intergeneric hybrid	[110]
Wheat (*T. aestivum* × *Psathyrostachys huashanica*)	Embryo culture	Resistance to powdery mildew	[111]
Wheat × rye (*T. aestivum* × *Secale cereale*)	Embryo rescue	Embryo lethality	[112]
Wheat × Rye (*T. aestivum* × *Secale cereale*)	--	Resistance to powdery mildew by translocation of 4R chromosome	[113]
Brassica (*Sinapis alba* × *Brassica juncea*)	Somatic hybridization through protoplast fusion	Resistance to *Alternaria brassicae* and heat stress	[114]
Brassica (*Sinapis alba* × *Brassica juncea*)	Somatic hybridization through protoplast fusion	Broaden genetic variation along with resistance to Alternaria brassicae	[115]
Brassica (*Brassica juncea* × *Sinapis alba*)	Somatic hybridization through protoplast fusion	Development of a yellow-seeded stable allohexaploid	[116]
**Doubled haploid**			
Durum wheat	Unpollinated ovary culture	Production of doubled haploid	[6]
Barley	Anther culture	Production transgenic homozygous DH lines	[117]
Barley	Anther culture	Haploid production	[118]
Lentil	Immature embryo culture	To shorten the breeding cycle	[119]
Wheat	Anther culture	Chromosome doubling	[120]
Wheat	Anther culture	Doubled haploid	[121]
Wheat	Microspore culture	Haploid production and resistance to *Gibberella zeae*	[122]
Lentil	Embryo rescue	Overcome reproductive barriers and hybrid recovery	[123]
Alloplasmic (*H. vulgare* × *T. aestivum*) and euplasmic line	Anther culture	Yield and quality traits, resistance to fungal diseases	[124]
**Somaclonal variation**			
Barley	Endosperm-supported mature embryo	Somaclonal variation	[125]
Spelt wheat	Anther and isolated microspore culture	Induces genetic variation	[126]
Barley	Immature zygotic embryo culture	To modify tissue culture-induced variation	[87]
Egyptian barley	Mature embryo culture	Somaclonal variation	[127]
Maize	Immature embryo culture	Genetic variation	[128]
Wheat	Somatic hybridization	Stem rust	[129]
Maize	Cell culture from an immature embryo	Epigenomic variation	[130]
**Mutation breeding**			
Wheat	Asymmetric somatic hybridization	Genome rearrangement, sequence elimination, and genetic variation via point mutations and indels	[131]
Wheat	Asymmetric somatic hybridization	Affects synonymous codon usage	[132]
Wheat	Asymmetric somatic hybridization	Induces genome-wide genetic variation	[133]
Wheat and maize	Immature embryo culture	Synthesis and study of a wheat/maize hybrid CENH3 gene	[134]

## 7. Application of Biotechnology 

In recent years, considerable progress has been made in the genetic engineering of monocotyledonous crop plants. However, the remaining bottlenecks in regenerating fertile plants from the single transformed cell are the predictability and reliability of foreign gene expression. The “one and only” method for cereal transformation does not yet exist. Many questions are still open concerning the frequency and quality of transformation. Some of the remaining challenges in gene transfer in cereals are controlling integrated copy numbers.

To produce a genetically modified plant, a foreign gene must be introduced into all the cells that make up the plant. In general, there are two types of techniques currently used. One is a method of transfecting a tissue piece or callus in the culture process and regenerating (redifferentiating) a plant from the obtained transformed cells. In such instances, tissue culture is indispensable for many crops to produce recombinants. The other is a planta transformation method in which genes are directly introduced into germ cells during the growth of plants to obtain gene recombinants in the next generation. However, the technical hurdles are high, and the only widely-used method is the floral dip method of *Arabidopsis thaliana*, which infects ovules with *Agrobacterium tumefaciens* [135].

### 7.1. Genome Editing 

Genetic engineering allows precision breeding that enables genetic variation (desired traits) in plants to drive new agricultural advancements. Various genetic engineering techniques have been utilized to generate improved crop plants. Transgenesis is the insertion of recombinant genetic elements in which one or more components (gene, promoter, and terminator) are taken from a sexually incompatible gene pool. Cisgenesis involves inserting an identical copy of a complete genetic element, including the gene, promoter, and terminator, within a sexually compatible gene pool. However, unlike cisgenes, intragenes are hybrid genes, and one or more components (gene, promoter, and terminator) of recombinant genetic elements isolated from different genes within a sexually compatible gene pool are inserted into the crop of interest. In recent years, modern genome editing methods viz. zinc-finger nucleases (ZFNs), transcription activator-like (TAL) effector nucleases (TALENs), and clustered regularly interspaced short palindromic repeats (CRISPR) associated with the Cas9 and Cpf1 proteins have allowed researchers to simply, swiftly, and inexpensively insert site-specific modifications into desired DNA sequences. These techniques have been implemented in gene editing in cereal crops, and some examples are shown in Table 2. These methods remove the limitations of conventional breeding methods, and recent reviews highlight different genome editing tools and potential applications in crop improvement in depth. A recent review presents an analysis of the current state of genome editing in the major cereal crops [136,137,138,139,140]. 

The CRISPR genome editing system has superseded its previous counterpart as capable of creating precise mutations in targeted genes, which can be tailored easily to target the desired locus to be edited. As a result, it has brought an unparalleled revolution in agricultural sciences and precision plant breeding.

#### 7.1.1. CRISPR/Cas9

The clustered regularly interspaced short palindromic repeats (CRISPR)-associated endonuclease Cas9 (CRISPR/Cas9) system from *Streptococcus pyogenes (SpCas9*) was the first well-characterized RNA-guided endonuclease to target a specific genomic sequence using an engineered 20 base pair (bp) RNA guide sequence that binds to matching DNA and the Cas9 protein, upon recognition of an additional 3′ localized protospacer adjacent motif (PAM) sequence 5′-NGG-3′, and to generate a double-strand break at a desired location in the genome. This genome editing method allows DNA insertion, deletion, or modification with high specificity and efficiency [159,160,161] For example, CRISPR/Cas9 increased the seed size in wheat by knocking out the function of all homeologs of *TaGW2*, a gene known as a negative regulator of seed size [162]. Similarly, transgene-free low-gluten wheat has recently been engineered with CRISPR/Cas9 [161]. However, CRISPR editing is challenging to perform in wheat as its complex genetic structure leads to high levels of off-target activity and genetic redundancy. Compared to other crops, such as rice, only a few studies are available to validate the CRISPR technology in wheat. Recent studies comprehensively reviewed the CRISPR-based genome editing in wheat [11,163].

#### 7.1.2. CRISPR/Cpf1 System

The nuclease Cas12a requires a small crRNA to induce double-strand breaks with efficiencies similar to CRISPR/Cas9. Moreover, this nuclease uses an 18–23 nucleotide spacer for maximum efficiency and specificity, identifies a T-rich PAM located 5′ upstream of the guide, and generates staggering ends with 5′ overhangs. Compared to Cas9, it suggests that Cpf1 orthologs, AsCpf1 (*Acidaminococcus spp.* BV3L6) and LbCpf1 (*Lachnospiraceae bacterium* ND2006), could be successfully used for targeted gene editing in wheat with minimal off-target effects [164,165].

#### 7.1.3. Base Editing

This system borrowed from CRISPR is a precise genome editing approach that allows the conversion of nucleotides without inducing double-stranded DNA breaks (DSB) or using donor templates. It is based on Cas9 nickase fusions to a nucleotide deaminase domain [166,167]. It has been used for changing a C–G base pair into T-A (cytidine deaminase base editor) or A–T into G-C (adenosine deaminase base editor) [168]. For example, the adenine base editors (ABE) system has been used to generate base-edited plants in wheat by targeting *TaDEP1* and *TaGW2* genes [169].

#### 7.1.4. Prime Editing

This system is also borrowed from the CRISPR system that uses a catalytically impaired Cas9 endonuclease to a reverse transcriptase enzyme and a prime editing guide RNA (pegRNA). This complex can identify a target site and replace the target DNA nucleotides without double-stranded DNA breaks or donor templates [170]. This enables all 12 types of base substitutions and small indels, substantially expanding the scope and capabilities of precise genome editing [171]. For example, prime editing has been used to generate base-edited plants in wheat by targeting *TaGW2*, *TaGASR7*, *TaDME1*, *TaLOX2*, and *TaMLO* [172].

### 7.2. Genetic Transformation

**Figure 2:**
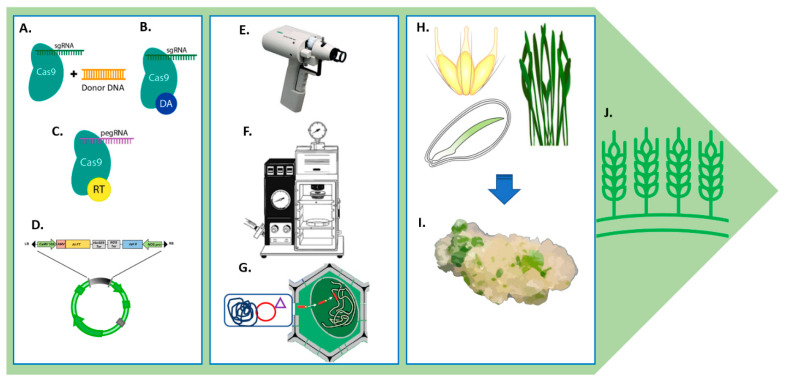
**Schematic overview of the genome editing methods for genome editing in wheat.** (**A–D**): show different types of genetic modifications generated by CRISPR-based genome editors. (**A**) CRISPR editing with double-stranded DNA breaks, (**B**) base editing, and (**C**) prime editing, (**D**) regular gene transformation cassette with selectable markers. (**E–G**): show plant genetic transformation approaches, biolistics, or particle bombardment methods. (**E**) Helios gene gun system, (**F**) PDS-1000/He system uses high-pressure helium gas to accelerate nucleic acid-coated gold or tungsten microparticles to velocities necessary to transfect cells, tissues, or organelles. (**G**) *A. tumefaciens* transformation with the Ti plasmid. (**H–I**): show various forms of explants (leaf, embryo, spikes) used to generate callus and transformations. (**J**) After gene transformation, tissues are followed by plant regeneration until the acclimation step and plant hardening before transfer to soil.

Plant genetic transformation allows the direct insertion of agronomically important genes into major crops. It is an essential tool in breeding programs for developing unique and genetically varied plants. The transferred gene is known as a “transgene,” and the organisms that result from a successful gene transfer are known as “transgenics” [173]. Several gene delivery systems use different approaches to introduce genetic components into viable host cells (Figure 2).

#### 7.2.1. Biological Systems

When a part of plant tissue (outer cell mass) is cultured in the presence of appropriate plant hormones, a cell called a callus is formed. These cells make up the explants dedifferentiated and proliferated, and each cell is undifferentiated. Therefore, selecting only transformed cells from the callus undergoing gene transfer treatment is possible, and regenerating a complete plant individually is possible. The redifferentiation ability of plants in tissue culture depends on the explants to be tested, and the selection is essential. Hypocotyls and leaf pieces are often used in dicotyledonous plants, but seed embryos and scutellum are often used to transform monocotyledonous plants, especially rice [174].

On the other hand, immature embryos in the early ripening stage are used in corn, wheat, barley, and sorghum crops. Immature embryos have excellent culture characteristics, but to obtain vigorous proliferative activity, the cultivation conditions up to the acquisition of immature embryos are essential, and sufficient attention must be paid. In addition, the redifferentiation ability in tissue culture differs significantly between cultivars. For example, a transformation system mediated by *Agrobacterium* has been established for rice even in essential cultivars such as “Koshihikari” and “Hitomebore” [175], and wheat embryos used for transformation and developed cultivars such as “Fielder” and “Bobwhite” [176].

*Agrobacterium*-mediated plant genetic transformation is one of the most successful and widely utilized biological systems. Compared to dicotyledonous plants, monocotyledonous plants have a lower infection efficiency of *A. tumefaciens*, making it difficult to infect plants directly. Herrera-Estrella and colleagues were the first to describe the generation of a transgenic plant using *A. tumefaciens* [177]. The high rate of gene transfer, effective transgene insertion into the host genome, and low copy number of the integrated foreign gene are significant benefits of *Agrobacterium*-mediated transformation [178]. Moreover, it can infect a wide range of dicot plants. However, until the mid-1990s, effective monocotyledon transformation by *A. tumefaciens* was impracticable because monocotyledons were considered beyond the host range of crown gall disease caused by *A. tumefaciens*. Therefore, it was believed that *A. tumefaciens* could not transform monocots. After years of experiments, this perspective was completely changed. Numerous species, including cereals [179,180,181,182,183], gymnosperms [184,185,186,187], and algae [188] that were previously recalcitrant to *A. tumefaciens* infection can now be transformed. *A. tumefaciens* is not the only bacterium tested for the biological transformation of plants. A few additional bacterial genera outside the *Agrobacterium* genus, such as *Rhizobium*, *Sinorhizobium*, and *Mesorhizobium* are capable of genetic transformation by acquiring a disarmed Ti plasmid binary vector [189].

Almost every genetic transformation process requires three critical elements: the susceptibility of the infected tissue to *Agrobacterium*, the capacity of the target tissue to regenerate, and an efficient screening system for the recovery of transformed plants [190]. The cereals’ resistance to all the above factors, especially to tissue culture and the regeneration of target tissues, has been a significant impediment to biotechnology applications in agronomically important crops such as wheat, which remains highly genotype-dependent. For example, a study showed that only three wheat cultivars (V-III-83, Faisalabad-2008, and AQS-11) out of 13 varieties tested for callus formation, transient GUS expression, and regeneration were comparatively better in tissue culture response and might be suitable for transformation [191]. However, a year later, Japan Tobacco Inc. claimed a significant advancement in *Agrobacterium*-mediated wheat transformation efficiency (PureWheat technology). This method is based on an optimized tissue culture medium and a transformant selection method tested on the spring wheat cultivar Fielder, which resulted in 50–90% transformation efficiency [176].

Furthermore, PureWheat technology has been tested on nine Australian commercial wheat varieties, where eight of them successfully transformed with transformation efficiencies ranging from 1.5 to 51% [192]. Wang and colleagues used a slightly modified PureWheat protocol to transform 15 varieties of commercial Chinese wheat, resulting in a transformation efficiency of up to 37.7% [193]. However, this strategy must be refined further to overcome the constraints of cultivars with high genotype dependence, such as Aikang58 and Jing411, and Janz [128,192,193].

Using regeneration-related genes during in vitro culture can improve plant transformation efficiency [128,194,195,196,197]. A recent study showed that overexpression of the wheat gene *TaWOX5* from the WUSCHEL family significantly boosts immature embryo transformation efficiency while being less genotype-dependent [128]. It did not impair shoot differentiation or root development, as reported in other cereal crops when morphogenic regulators such as WUS2 and BBM were overexpressed [196].

Not only the morphogenic regulators, but also growth regulating factors (GRFs) alone or in chimeras with GRF-interacting factor (GIF) could enhance regeneration from tissue culture [198]. The transgenic expression of wheat GRF4–GIF1 chimera significantly increased the regeneration efficiencies of durum wheat, common wheat, rice, and triticale. Transgenic plants overexpressing the GRF4–GIF1 chimera were fertile and displayed normal phenotypes. Furthermore, GRF4–GIF1 promotes effective wheat regeneration without exogenous cytokinins, which aids in selecting transgenic plants without selectable antibiotic markers [199]. The GRF–GIF has been an ideal system for extending genome editing techniques such as CRISPR/Cas9 to crops with low regeneration efficiencies. This concept proved, and the study recovered, 30 independent transgenic lines, which showed Cas9-induced editing through the disruption of the *StyI* restriction site [199]. The edited T_0_ plants were fertile and showed mutant *q* null phenotypes as the Cas9 targeted the wheat *Q* gene.

#### 7.2.2. Non-Biological Systems

The biolistic wheat transformation has become a popular method for producing transgenic wheat plants. It has been less genotype-dependent and, in most cases, more efficient than *Agrobacterium*-mediated methods. The trait gene is co-bombarded with a distinct selectable marker plasmid, making it less demanding in its vector requirements. The DNA integration events, on the other hand, are generally more complex and contain more transgene copies [200,201]. Random multi-copy transgene integration is the key challenge of stable biolistic transformation. It frequently results in abnormal inverted repeat compositions or transgene rearrangements [202]. This could lead to transgenic silencing [200,203], deletion [204], and abnormal transgene expression in successive generations [205,206]. The effectiveness of wheat biolistic transformation is poor and mostly ranges from 1–5%: insufficient for the large production of fertile transgenic plants [207,208]. However, another study obtained 3.1–20.3% transformation efficiency with the commercial wheat cultivar Gladius by using polyethyleneglycol (PEG) and magnesium salt solutions to precipitate the DNA onto gold microparticles instead of spermidine and calcium chloride as in the usual coating approach [202].

To avoid the problems associated with tissue culture and regeneration procedures, *in planta* techniques that transfer foreign genes directly into intact plant tissue have been established in a variety of plant species such as *Arabidopsis*, *Medicago truncatula*, tomato, and a few monocots such as maize, rice, and wheat [209,210,211,212,213]. In wheat, microprojectile-mediated DNA transfer to the shoot apical meristems grown from dry imbibed seeds produced stably transformed transgenic wheat plants in an experimental cultivar (Fielder) and an elite commercial cultivar (Haruyokoi). In one successful study of *the planta transformation method using particle bombardment (iPB), undifferentiated germ cells from the apex tissue (L2 layer) were* used as the target. If DNA can be introduced into the L2 layer by particle bombardment, stable transformants inherited to the next generation can be obtained. Due to the physical gene transfer method, it is considered that the dependence on plant species and varieties is low [210]. With the iPB genome editing technology, targeted mutations can be introduced into germ cells in the shoot apex. A recent study shows that *the TaGASR7 gene (a member of the Snakin/GASA gene family)* involved in grain length and weight was CRISPR/Cas9 edited, and the cassette was transferred via the iPB [214].

## 8. Discussion

Some of the remaining challenges in gene transfer in cereals are the control of integrated copy number, gene targeting, plastid transformation, inheritance of stable expression over many generations, inducible and tissue-specific expression, quality and persistence of selective marker genes, and still, in most cereal species, the low transformation frequency. This low frequency is primarily due to the limiting regeneration potential. Due to the simplicity of design and high efficiency of DNA cleavage, the CRISPR/Cas9 system is rapidly becoming widespread, and genome editing technology is becoming a general purpose tool used in individual laboratories. It is also possible to select the optimal artificial restriction enzyme according to the target DNA sequence, such as TALEN, which has a less off-target effect, and SaCas9. Cpf1 (Cas9-like protein) recognizes PAM sequences different from SpCas9. In addition, directly introducing the artificial restriction enzyme gene into the shoot apex in the seed embryo makes it possible to perform genome editing in practical varieties without using callus culture. Therefore, it is expected to develop in practical varieties of crops other than wheat. Recently, a technique for introducing RNP (ribonucleoprotein), a complex of Cas9 protein and gRNA, into protoplasts and immature embryos and performing genome editing has also been reported [215,216]. 

To improve wheat breeding, we have to interfere with the system directly. This requires that we understand the causalities on a molecular level. The increased accessibility and quantity of genomic sequence data enable the generation of novel hypotheses for understanding plant genomes. Gene editing makes it possible to test hypotheses to answer how individual genomes behave in a complex environment since phenotype causality should also differ between individuals. By this, we can answer what the relationships between crops and their wild relatives are and how various population genetic parameters vary across species. This insight can help improve our understanding of how plants may adapt to future climate change by studying how evolutionary changes occur at the genetic, chromosomal, individual, and population levels as before in wheat. Plant tissue culture enables a fast gene editing process, and novel plant varieties can be tested in different environments to ensure efficiency in different geographic locations and growing conditions in a shorter time frame. Acquiring the respective knowledge will be the main challenge in the future. Therefore, genome editing will accelerate breeding progress along with the tissue culture, molecular plant breeding, and efforts within crop-based communities and multidisciplinary crosstalk. Hence, *in planta* genome editing methods will significantly contribute to the social implementation of genome-edited crops to innovate the breeding pipeline and leverage unique climate adaptations. In addition, societal expectations of modern agribusiness demand a decreased dependence on chemicals and increased stewardship of land and water resources, conserving them for future generations. With climate change affecting agriculture and food systems, we have new scientific innovations that can be deployed to mitigate the effects and reduce the impact of food production on the environment.

## Figures and Tables

**Table 2:** List of some significant gene editing studies in cereal crops.

Crop	Genome Editing Technique	Targeted Gene	Molecular Function	Type of Editing	Effect	Reference
Wheat	ZFN	AHAS	Role in branched amino acid formation	Insertion and replacement	Resistance against herbicide	[141]
	ZFN	IPK1	Phytate formation	Deletion	Removal of antinutritional phytate, mineral accumulation against abiotic stress (Fe, Zn)	[142]
	CRISPR/Cas9	IPK1	Phytic acid biosynthesis	Deletion and insertion	Reduced phytic acid and enhanced Fe and Zn in wheat grains	[143]
	CRISPR/Cas9	HRC	Encodes a putative histidine-rich calcium-binding protein	Deletion and insertion	Reduced fusarium head blight severity	[144]
	CRISPR/Cas9	GW2	Genetic determinant of grain weight	Deletion	Increase in thousand-grain weight and grain protein content	[145]
Rice	ZFN	SSIVa	Involved in the starch biosynthesis pathway	Deletion	Improve eating quality	[146]
	TALENs	11N3	Rice bacterial blight susceptibility gene	Deletion and insertion	Increase resistance to rice bacterial blight	[147]
	CRISPR/Cas9	SAPK2	Regulate drought response	Deletion	Improved drought tolerance	[148]
	CRISPR/Cas9	OsPIN5b GS3 OsMYB30	Regulate panicle length, grain size, and cold tolerance	Mutation deletion	Increased panicle length, enlarged grain size, and increased cold tolerance	[149]
	CRISPR/Cas9	NRT1.1B	Control yield and early maturation	Base editing	Increased yield	[150]
Maize	ZFN	IPK1	Catalyses the final step in phytate biosynthesis in maize seeds	Deletion and insertion	Removal of antinutritional phytate herbicide tolerance	[151]
	TALENs	gl2	Cuticular lipid functions	Deletion	Reduce epicuticular wax	[152]
	CRISPR/Cas9	RR22	Salinity tolerance	Deletion, insertion, substitution	Regulation of salt tolerance	[153]
	CRISPR/Cas9	NC1 QTL and, HKT1	Encodes an HKT-type transporter	Deletion	Reduce salt tolerance	[154]
	CRISPR/Cas9	AOC	Jasmonic acid biosynthesis pathway	Deletion, insertion, substitution	Efficient coordination with the environment	[155]
Barley	CRISPR/Cas9	HPT HGT	Biosynthesis of tocotrienols and tocopherol	Deletion and insertion	Decreased grain size and weight Shrunken phenotype Lower total starch content in grains	[156]
		ARE1	Involved in nitrogen use efficiency	Missense and/or frameshift mutations	Increase in plant height, tiller number, grain protein content, yield, and chlorophyll content	[157]
	CRISPR/Cas9	ENGase	Production of GN1 type FNGs (Free N Glycans	Indels and deletions	Increased abiotic tolerance	[158]
